# Pseudomyxoma peritonei presenting with pyloric obstruction: a case report

**DOI:** 10.1093/jscr/rjag516

**Published:** 2026-06-26

**Authors:** Eyad Jamileh, Zuha Akhtar, Maria Babu

**Affiliations:** School of Medicine, Queens University Belfast, 97 Lisburn Road Belfast, BT9 7BL, United Kingdom; Department of Gastroenterology, Royal Blackburn Teaching Hospital, Haslingden Road, East Lancashire Hospitals Trust, Blackburn, BB2 3HH, United Kingdom; School of Medicine, University of Oxford, Wellington Square, Oxford, Oxfordshire, OX1 2JD, United Kingdom; Department of Medicine, Royal Preston Hospital, Sharoe Green Lane, Lancashire Teaching Hospitals NHS Foundation Trust, Preston, PR2 9HT, United Kingdom

**Keywords:** pseudomyxoma peritonei, gastric outlet obstruction, extrinsic compression, duodenal stenting, endoscopic gastrojejunostomy

## Abstract

Pseudomyxoma peritonei (PMP) is a rare malignancy characterized by mucinous ascites. Gastric outlet obstruction (GOO) due to extrinsic compression is exceptionally uncommon. We present a 76-year-old male with high-grade appendiceal mucinous neoplasm presented with progressive abdominal pain, nausea, and vomiting. Imaging demonstrated extensive peritoneal disease causing pyloroduodenal compression. Five sequential self-expanding metal stents were placed, providing only transient relief due to persistent compression and tumour overgrowth. With worsening nutritional status, a multidisciplinary team pursued a palliative approach. An endoscopic gastrojejunostomy was performed alongside total parenteral nutrition and nasogastric decompression, resulting in sustained symptom relief and improved quality of life. PMP-related GOO poses significant management challenges. Endoscopic stenting may be ineffective in diffuse mucinous disease. Early consideration of bypass procedures, such as gastrojejunostomy, combined with multidisciplinary care and nutritional optimization, is essential for durable palliation.

## Introduction

Pseudomyxoma peritonei (PMP) is a rare malignancy characterized by the accumulation of gelatinous mucinous tumour implants within the peritoneal cavity [1]. Its incidence is ~3.2 per million per year, with an estimated prevalence of 22 per million [2]. PMP most commonly arises from a ruptured appendiceal mucinous neoplasm, although other primaries have been reported [3]. Chronic inflammation and genetic mutations, particularly KRAS and GNAS, are implicated in its pathogenesis [4].

Metastatic spread beyond the peritoneum is uncommon; instead, disease dissemination follows peritoneal fluid dynamics, with mucin accumulating in the omentum, paracolic gutters, and subdiaphragmatic regions [5]. Haematogenous or lymphatic spread is atypical and usually associated with high-grade disease [6]. Gastric outlet obstruction (GOO) due to extrinsic compression by mucinous implants is an unusual complication [7, 8].

PMP shows a slight female predominance [9], but this report highlights a rare presentation of GOO in a male patient. We describe the case and discuss the associated diagnostic and therapeutic challenges. The CARE checklist was completed (Supplementary material).

## Case presentation

A 76-year-old man with known PMP presented with worsening upper abdominal pain, distension, nausea, bilious vomiting, and increased ileostomy output, consistent with GOO. His history included a laparoscopic appendectomy 3 years prior for perforated appendicitis, with histology showing no mucinous neoplasia, suggesting a previously subclinical or subsequently developed appendiceal tumour. One year before presentation, he required a loop ileostomy for partial small bowel obstruction, likely secondary to undiagnosed PMP. His comorbidities included dyspepsia and benign prostatic enlargement.

Five months prior, contrast-enhanced computed tomography (CT) demonstrated extensive low-attenuation ascites with characteristic scalloping of the liver and spleen, and a 13 cm loculated collection in the right paracolic gutter, consistent with gelatinous ascites in PMP (Fig. 1). Exploratory laparotomy confirmed high-grade appendiceal mucinous neoplasm. Cytoreductive surgery was attempted but deemed incomplete due to extensive disease, and the patient was commenced on palliative FOLFOX chemotherapy [10], which was poorly tolerated.

**Figure 1:**
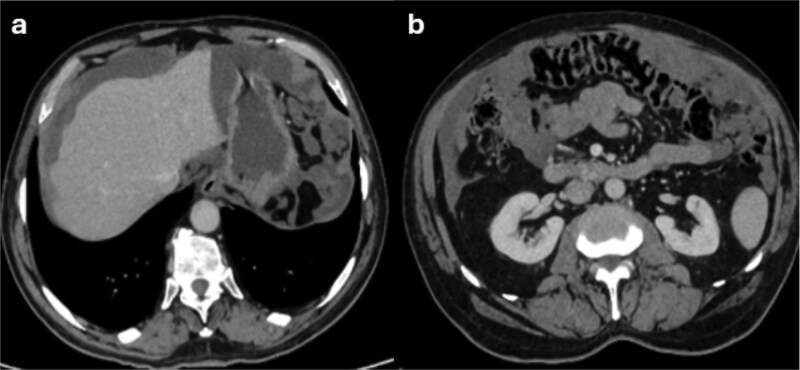
Contrast-enhanced CT of the abdomen demonstrating features of pseudomyxoma peritonei. (a) Extensive low-attenuation mucinous ascites throughout the peritoneal cavity causing mass effect with scalloping of the liver and splenic surfaces. There is near-complete encasement of intra-abdominal organs, including the stomach, spleen, omentum, and colon, extending into the pelvis-features typical of gelatinous ascites in PMP. (b) Large loculated mucinous collection within the right paracolic gutter, with the appendix as the likely primary source.

Following clinical deterioration, repeat CT showed a markedly distended stomach with an air–fluid level and abrupt narrowing at the pylorus and proximal duodenum due to extrinsic compression from peritoneal tumour deposits (Fig. 2). Oesophagogastroduodenoscopy (OGD) confirmed a slit-like narrowing at the pylorus and proximal duodenum (D2) with preserved mucosa, consistent with functional obstruction from external compression (Fig. 3). Given the patient’s frailty, an endoscopic approach was favoured.

**Figure 2:**
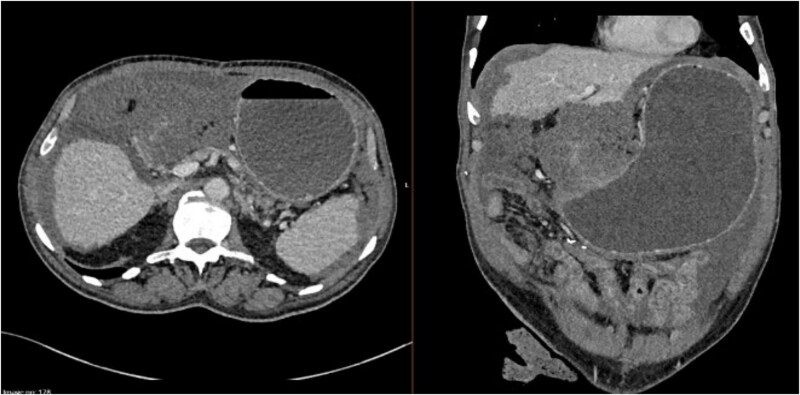
Contrast-enhanced CT of the abdomen and pelvis demonstrating gastric outlet obstruction secondary to pseudomyxoma peritonei. There is moderate-volume mucinous ascites with scalloping of the liver and associated peritoneal nodularity. The stomach is markedly distended with retained contents, consistent with gastric outlet obstruction due to extrinsic compression from peritoneal tumour deposits. No distal mechanical small bowel obstruction is identified.

**Figure 3:**
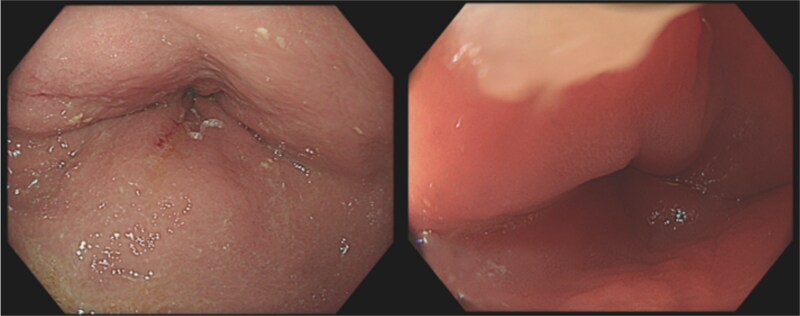
Endoscopic view demonstrating pyloric obstruction due to extrinsic compression. There is marked narrowing of the pyloric channel with a slit-like lumen, without evidence of an intrinsic mass lesion, consistent with external compression from mucinous peritoneal disease.

Over several weeks, five sequential duodenal self-expanding metal stents were placed using both covered and uncovered designs. Although transient symptom relief was achieved, obstruction recurred due to persistent extrinsic compression. Repeat endoscopy demonstrated marked mucosal hyperplasia at the proximal stent margin, with nodular, erythematous mucosa, raising the possibility of reactive change or tumour overgrowth (Fig. 4). Biopsy was not obtained; however, clinical and radiological findings strongly supported extrinsic tumour compression.

**Figure 4:**
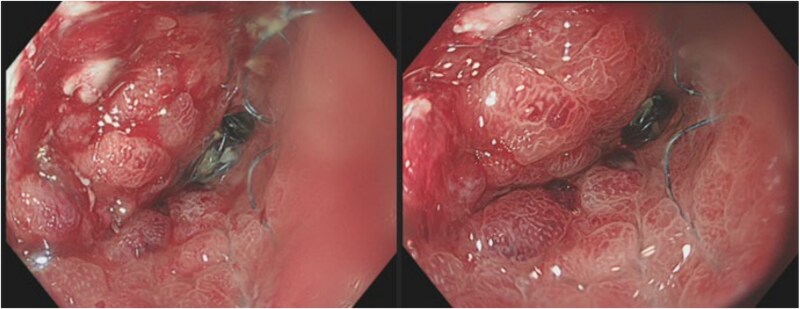
Endoscopic image following duodenal stent placement demonstrating persistent obstruction. The pyloric outlet shows a self-expanding metal stent in situ; however, luminal narrowing persists due to ongoing extrinsic compression. The surrounding mucosa appears erythematous and hyperplastic, suggestive of reactive change, although tumour overgrowth or ingrowth cannot be excluded.

Stent failure was likely multifactorial, including inadequate initial stent length and progressive mucinous tumour burden. A contrast study confirmed persistent GOO. Given worsening nutritional status, further stenting was abandoned following multidisciplinary discussion.

An endoscopic gastrojejunostomy was performed using a lumen-apposing metal stent (LAMS) under combined endoscopic and fluoroscopic guidance, creating a gastrojejunal bypass. A PEG tube was inserted for gastric decompression, and total parenteral nutrition (TPN) was initiated. This approach successfully relieved symptoms, improved nutritional status, and allowed transition to palliative care with a focus on symptom control.

## Discussion

PMP is an indolent but progressive disease often diagnosed late due to nonspecific early symptoms [11]. It is frequently identified incidentally or at advanced stages [12]. Common features include abdominal distension and discomfort, with bowel obstruction occurring as disease progresses [13]. While lower gastrointestinal obstruction is more typical, upper gastrointestinal involvement is rare [14].

GOO in PMP results from extrinsic compression by mucinous implants. Clément-Pastier *et al*. described how tumour accumulation around the lesser sac and duodenum can produce this effect [15]. Our findings are consistent with this mechanism.

Endoscopic duodenal stenting is widely used for malignant GOO due to its minimally invasive nature and rapid symptom relief [7]. However, its effectiveness is limited in PMP. The diffuse nature of mucinous disease leads to stent dysfunction, migration, or re-occlusion. In this case, repeated stenting provided only temporary benefit, consistent with studies demonstrating limited long-term efficacy in carcinomatosis-related GOO [16].

Surgical gastrojejunostomy offers more durable relief but carries significant risk in frail patients. Endoscopic gastrojejunostomy has emerged as a less invasive alternative, with favourable outcomes [16]. In this patient, it provided sustained palliation after failure of stenting.

This case highlights the importance of early multidisciplinary team involvement, which may reduce repeated interventions and expedite definitive management. Nutritional support is essential, as persistent vomiting predisposes to malnutrition and electrolyte disturbances. While enteral approaches are often poorly tolerated [17], TPN may provide stabilization despite associated risks, and early dietetic input is recommended [18].

A limitation of this case was the absence of biopsy from the hyperplastic mucosa. Although findings supported extrinsic compression, histological confirmation would have increased diagnostic certainty.

In advanced PMP, aggressive interventions such as repeated stenting or incomplete cytoreduction may offer limited benefit. Evidence suggests incomplete cytoreductive surgery confers little survival advantage in patients with high tumour burden [19], while chemotherapy may provide modest disease control but is often poorly tolerated [10, 20].

This case is unique in demonstrating refractory GOO in PMP requiring multiple sequential stenting attempts before definitive palliation. It highlights key learning points: extrinsic compression should be suspected when mucosa is preserved endoscopically; repeated stenting in diffuse mucinous disease offers diminishing returns; and early consideration of bypass strategies, particularly endoscopic gastrojejunostomy, may provide more durable relief. Ultimately, timely MDT involvement, proactive nutritional support, and prioritization of quality of life are essential. Clearer prognostic tools incorporating tumour burden and biomarkers are needed to guide decision-making and support timely transition to patient-centred palliative care.

## Supplementary Material

care_checklst_rjag516
