# Supplementary material for: Pseudomyxoma peritonei presenting with pyloric obstruction: a case report
Source: J Surg Case Rep. 2026 Jun 26;2026(6):rjag516. doi: 10.1093/jscr/rjag516 (PMC13308960; doi:10.1093/jscr/rjag516)
Supplement: care_checklst_rjag516 [file care_checklst_rjag516.docx]

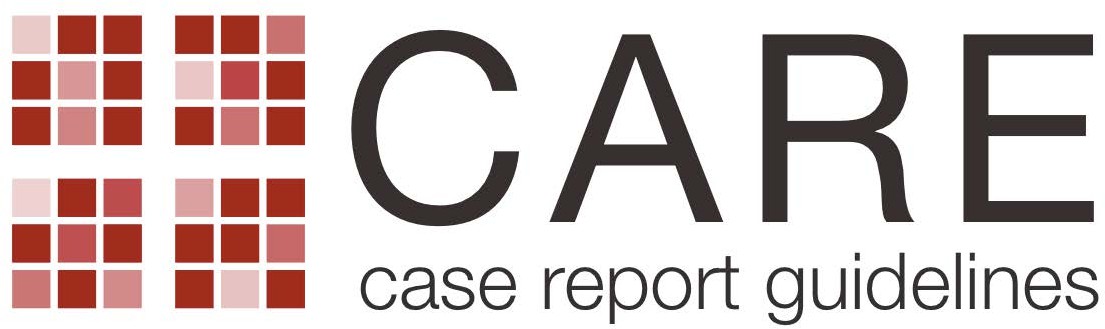
CARE Checklist of information to include when writing a case report
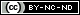


**Topic Item Checklist item description Reported on Line**

**Title 1** The diagnosis or intervention of primary focus followed by the words “case report” . . . . . . . . . . . . . . . . . . 1

**Key Words 2** 2 to 5 key words that identify diagnoses or interventions in this case report, including "case report" 6

**Abstract**

**(no references)**

**3a** Introduction: What is unique about this case and what does it add to the scientific literature? 9

**3b** Main symptoms and/or important clinical findings . . . . . . . . . . . . . . . . . . . . . . . . . . . . . . . . . . . . . . . . . . . . . . . . . . . 14

**3c** The main diagnoses, therapeutic interventions, and outcomes 22

**3d** Conclusion—What is the main “take-away” lesson(s) from this case? 27

**Introduction 4** One or two paragraphs summarizing why this case is unique (**may include** reference**s**) 36

**Patient Information 5a** De-identified patient specific information 36

**5b** Primary concerns and symptoms of the patient 58

**5c** Medical, family, and psycho-social history including relevant genetic information 64

**5d** Relevant past interventions with outcomes 60

**Clinical Findings**

**Timeline**

**Diagnostic Assessment**

**Therapeutic Intervention**

**Follow-up and Outcomes**

1. Describe significant physical examination (PE) and important clinical findings 74
2. Historical and current information from this episode of care organized as a timeline 70

**8a** Diagnostic testing (such as PE, laboratory testing, imaging, surveys). 71

**8b** Diagnostic challenges (such as access to testing, financial, or cultural) 84

**8c** Diagnosis (including other diagnoses considered) 74

**8d** Prognosis (such as staging in oncology) where applicable n/a

**9a** Types of therapeutic intervention (such as pharmacologic, surgical, preventive, self-care) . . . . . . . . . . . . . . . . . 91

**9b** Administration of therapeutic intervention (such as dosage, strength, duration) 91

**9c** Changes in therapeutic intervention (with rationale) 99

**10a** Clinician and patient-assessed outcomes (if available) 127

**10b** Important follow-up diagnostic and other test results 127

**10c** Intervention adherence and tolerability (How was this assessed?) 124

**10d** Adverse and unanticipated events 126

**Discussion 11a** A scientific discussion of the strengths AND limitations associated with this case report 130

**11b** Discussion of the relevant medical literature **with references** 132

**11c** The scientific rationale for any conclusions (including assessment of possible causes) 148

**11d** The primary “take-away” lessons of this case report (without references) in a one paragraph conclusion 209

**Patient Perspective 12** The patient should share their perspective in one to two paragraphs on the treatment(s) they received . . . . Na

**Informed Consent 13** Did the patient give informed consent? Please provide if requested . . . . . . . . . . . . . . . . . . . . . . . . . . . . . . . . . . . . . . **Yes
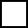
** **~~No~~
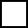
**
